# Type III Secretion in the Melioidosis Pathogen *Burkholderia pseudomallei*

**DOI:** 10.3389/fcimb.2017.00255

**Published:** 2017-06-15

**Authors:** Charles W. Vander Broek, Joanne M. Stevens

**Affiliations:** The Roslin Institute and Royal (Dick) School of Veterinary Studies, University of EdinburghMidlothian, United Kingdom

**Keywords:** T3SS, effector, translocator, *Burkholderia pseudomallei*, melioidosis

## Abstract

*Burkholderia pseudomallei* is a Gram-negative intracellular pathogen and the causative agent of melioidosis, a severe disease of both humans and animals. Melioidosis is an emerging disease which is predicted to be vastly under-reported. Type III Secretion Systems (T3SSs) are critical virulence factors in Gram negative pathogens of plants and animals. The genome of *B. pseudomallei* encodes three T3SSs. T3SS-1 and -2, of which little is known, are homologous to Hrp2 secretion systems of the plant pathogens *Ralstonia* and *Xanthomonas*. T3SS-3 is better characterized and is homologous to the Inv/Mxi-Spa secretion systems of *Salmonella* spp. and *Shigella flexneri*, respectively. Upon entry into the host cell, *B. pseudomallei* requires T3SS-3 for efficient escape from the endosome. T3SS-3 is also required for full virulence in both hamster and murine models of infection. The regulatory cascade which controls T3SS-3 expression and the secretome of T3SS-3 have been described, as well as the effect of mutations of some of the structural proteins. Yet only a few effector proteins have been functionally characterized to date and very little work has been carried out to understand the hierarchy of assembly, secretion and temporal regulation of T3SS-3. This review aims to frame current knowledge of *B. pseudomallei* T3SSs in the context of other well characterized model T3SSs, particularly those of *Salmonella* and *Shigella*.

## Introduction

Bacteria are required to adapt to and survive in constantly changing and harsh environments. In order to respond to and alter their environment, secretion systems have evolved in bacteria to export proteins into the surrounding milieu (reviewed in Costa et al., 2015). One secretion system in Gram-negative bacteria that has been the focus of much research in the last three decades is the Type III secretion system (T3SS) (reviewed in Galán et al., 2014). Type III Secretion Systems (T3SSs) have been shown to be important for virulence in many Gram-negative bacterial pathogens of animals and plants; including *Pseudomonas syringae, Xanthomonas, Ralstonia solanacearum, Erwinia*, pathogenic *Escherichia coli, Salmonella, Shigella, Yersinia*, and *Burkholderia* (Gemski et al., 1980; Maurelli et al., 1985; Galán and Curtiss, 1989; Jarvis et al., 1995; Stevens et al., 2002; Büttner and He, 2009). T3SSs span the bacterial inner and outer membranes forming a “molecular syringe” which allows bacteria to export proteins, called effectors, from the bacterial cytoplasm into a target eukaryotic cell (reviewed in Galán et al., 2014).

The focus of this review is T3SSs in the pathogenic bacterium *Burkholderia pseudomallei*, and to some extent the closely related species *B. mallei* and *B. thailandensis*. Originally described in drug addicts in Rangoon in the early twentieth century by Alfred Whitmore (Whitmore, 1913), *Burkholderia pseudomallei* is a facultative intracellular pathogen (Pruksachartvuthi et al., 1990) that is the causative agent of melioidosis, or Whitmore's disease. Melioidosis is a severe disease of humans and animals, causing an estimated 165,000 cases of human melioidosis per year resulting in a predicted 89,000 deaths (Limmathurotsakul et al., 2016). Infection with *B. pseudomallei* is usually associated with environmental exposure and can occur through breaks in the skin, inhalation or ingestion (reviewed in Cheng and Currie, 2005). In the majority of cases, the incubation period for melioidosis is between 1 and 21 days following infection (Ngauy et al., 2005). About 50% of melioidosis cases affect people with diabetes and other important risk factors include lung disease, cystic fibrosis and excessive alcohol consumption (Currie et al., 2010). There are varied clinical presentations of *B. pseudomallei* infection ranging from skin infections to pneumonia and septic shock, which hampers accurate diagnosis in a clinical setting (Currie et al., 2010). *B. pseudomallei* is reported to be able to reactivate after remaining latent following a primary infection. The longest reported period between infection and reactivation occurred in a World War II veteran who manifested symptoms 62 years after exposure (Ngauy et al., 2005). *B. pseudomallei* has been classified as a bioterrorism agent by both the UK government and the US Centres for Disease Control and Prevention (reviewed in Rotz et al., 2002; Cheng and Currie, 2005).

*B. mallei*, the causative agent of glanders in horses and other solipeds, is a zoonotic pathogen with restricted host range (Yabuuchi et al., 1992; Srinivasan et al., 2001). In humans, *B. mallei* causes a disease similar to melioidosis and has been similarly classified as a potential bioterrorism agent in the UK and US (Rotz et al., 2002; Van Zandt et al., 2013). The soil saprophyte *B. thailandensis* is non-pathogenic and present in high numbers in the soils and standing waters of endemic areas. This species is commonly used as an alternative model system for *B. pseudomallei* and *B. mallei* studies as its genome encodes many homologs of virulence factors from these pathogenic species (Brett et al., 1998; Moore et al., 2004; Yu et al., 2006; Haraga et al., 2008).

## Type three secretion systems in *B. pseudomallei*

The *B. pseudomallei* genome encodes three T3SSs which are referred to as T3SS-1, T3SS-2, and T3SS-3. The genome of *B. pseudomallei* consists of two circular chromosomes, with all three T3SSs residing on chromosome 2 (Holden et al., 2004). T3SS-2 and T3SS-3 are present in the genomes of *B. mallei* and *B. thailandensis*, whereas T3SS-1 is absent from both (Rainbow et al., 2002). T3SS-1 and T3SS-2 are relatively poorly characterized and share homology with the Hrp2 family of T3SSs found in plant pathogens (Winstanley et al., 1999; Rainbow et al., 2002). The best characterized of the *B. pseudomallei* T3SSs, T3SS-3, is also known as the B*urkholderia* secretion apparatus (Bsa) T3SS. It is a member of the Inv-Mxi-Spa family of T3SSs from *Salmonella* spp. (SPI-1) and *Shigella flexneri* (Attree and Attree, 2001; Stevens et al., 2002; Egan et al., 2014).

*Burkholderia pseudomallei* T3SS-1 (BPSS1390-BPSS1410) and T3SS-2 (BPSS1610-BPSS1629) show closest homology to the Hrp2 T3SS of the plant pathogen *Ralstonia solanacearum* (Angus et al., 2014). *B. pseudomallei* T3SS-2 expression is activated by the AraC-type regulator HrpB (BPSS1610) (Lipscomb and Schell, 2011). HrpB also regulates the expression of a type IV pilus encoded directly upstream of T3SS-2, but does not appear to regulate the other T3SSs in *B. pseudomallei* (Lipscomb and Schell, 2011). In order to investigate the role that T3SS-1 and T3SS-2 play in plants, a tomato plant infection model was established for *B. pseudomallei* and *B. thailandensis* (Lee et al., 2010). *B. pseudomallei* KHW T3SS-1 and T3SS-2 mutants were reported to be attenuated in tomato plants (Lee et al., 2010). However, in a more recent study, *B. thailandensis* did not display phytopathogenic activity in tomato plants which were treated identically to those in Lee et al. (2010), Lipscomb and Schell (2011). The question of whether *B. pseudomallei* is capable of infecting plants, and what if any role T3SS-1 and -2 have in this process, remain important unanswered questions.

The function of T3SS-1 and -2 in mammalian systems has also been investigated to some extent. T3SSs-1 and -2 do not appear to be required for vacuole escape of the bacterium into the cytoplasm of infected macrophages (Burtnick et al., 2008), and are dispensable in a Syrian hamster model of infection (Warawa and Woods, 2005). However, a *B. pseudomallei* T3SS-1 mutant displayed increased co-localisation with the autophagy marker LC3 and a reduction in intracellular survival in RAW264.7 cells (D'Cruze et al., 2011). In the same study, T3SS-1 was required for full virulence in a respiratory murine model of melioidosis (D'Cruze et al., 2011). Both earlier studies inactivated T3SS-1 by mutating the structural auto-protease component (BpscU, Table 1) (Warawa and Woods, 2005; Burtnick et al., 2008) while the latter study generated a system knockout by mutation of the ATPase (BpscN, Table 1), which may account for the different phenotypes observed.

**Table 1:** *B. pseudomallei* T3SS-1 and -2 genes, corresponding proteins and predicted functions.

***B. pseudomallei*** **K96243**		***B. pseudomallei*** **K96243**		***R. solanacearum***	**Universal Nomenclature**	**Predicted function**
**T3SS-1**		**T3SS-2**		**Hrp2**	**Protein name**	
**Locus tag**	**Protein name**	**Locus tag**	**Protein name**	**Protein name**		
BPSS1388	Similar to Hrpk1 from *P. syringae*					Translocator
BPSS1390	BpscC	BPSS1592	BpscC2	HrcC	SctC	Outer membrane ring / secretin
BPSS1391	BpspB	BPSS1610	BpspB2	HrpB		T3SS Regulator
BPSS1392	BpscT	BPSS1629	BpscT2	HrcT	SctT	Inner membrane export apparatus
BPSS1393	BpspD	BPSS1628	BpspD2	HrpD	SctO	Stalk protein
BPSS1394	BpscN	BPSS1627	BpscN2	HrcN	SctN	ATPase
BPSS1395	BpscL	BPSS1626	BpscL2	HrcL	SctL	Stator protein
BPSS1396	BpspH	BPSS1625	BpspH2	HrpH		Inner membrane component
BPSS1397	BpscJ	BPSS1624	BpscJ2	HrcJ	SctJ	Inner membrane ring component
BPSS1398	BpspJ	BPSS1623	BpspJ2	HrpJ		Inner rod component
BPSS1399	BpspK	BPSS1622	BpspK2	HrpK		Inner rod component
BPSS1400	BpscU	BPSS1621	BpscU2	HrcU	SctU	Autoprotease, early/middle substrate switch
BPSS1401	BpscV	BPSS1620	BpscV2	HrcV	SctV	Export gate
BPSS1402	BpsaP	BPSS1619	BpsaP2	HpaP	SctP	Regulates needle length / translocator secretion
BPSS1403	BpscQ	BPSS1618	BpscQ2	HrcQ	SctQ	Cytoplasmic ring
BPSS1404	BpscR	BPSS1617	BpscR2	HrcR	SctR	Inner membrane export apparatus
BPSS1405	BpscS	BPSS1616	BpscS2	HrcS	SctS	Inner membrane export apparatus
BPSS1406	BpspV	BPSS1615	BpspV2	HrpV		Secreted regulator
BPSS1407	BpscD	BPSS1614	BpscD2	HrcD	SctD	Inner membrane ring component
BPSS1410	BpsaB	BPSS1611	BpsaB2	HpaB		Chaperone

*Homologs from R. solanacearum Hrp2 T3SS are given for reference. Where applicable, the universal nomenclature for T3SS structural proteins is also listed to allow for comparison to genes from T3SS-3 listed in Table 2*.

### T3SS-3

**Figure 1:**
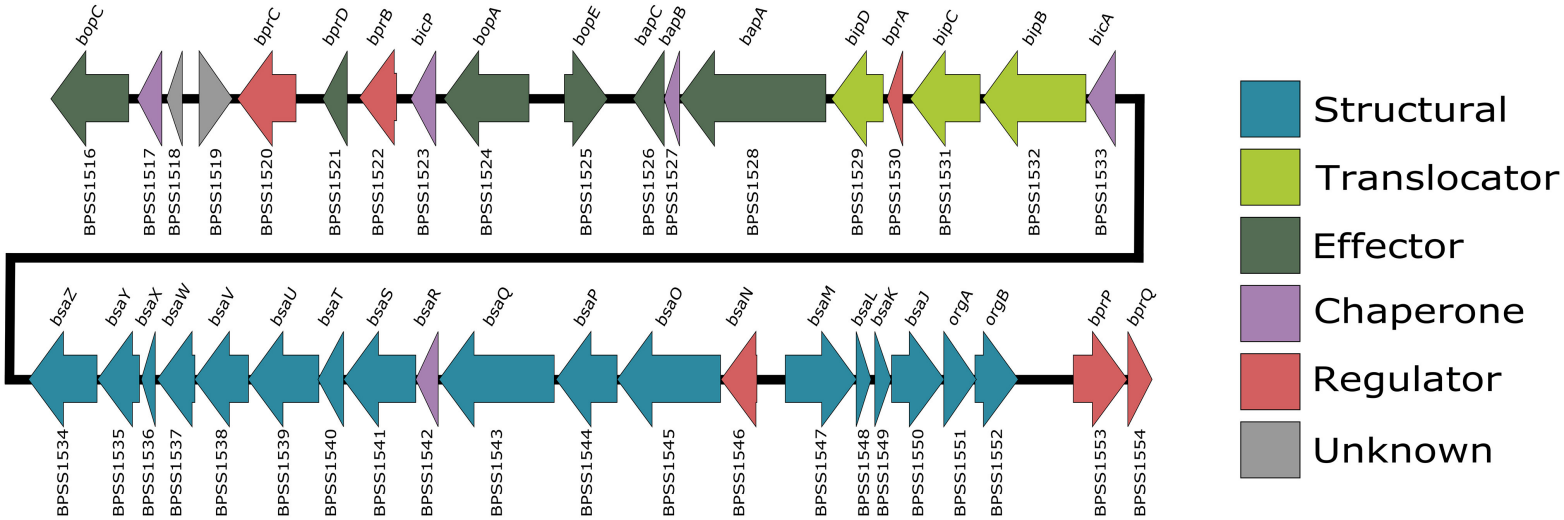
*B. pseudomallei* K96243 T3SS-3 gene locus. Also known as the *bsa* locus, the T3SS-3 genes are encoded by chromosome 2 (BPSS1516-BPSS1554). Arrows which point right represent genes encoded on the forward strand and arrows which point left represent genes encoded on the reverse strand. Gene locus tags are listed below each arrow and gene names are listed above. The arrows are color coded according to their predicted function.

The best characterized of the three T3SSs, T3SS-3 (BPSS1516-BPSS1552, Figure 1) is a member of the Inv/Mxi-Spa family of T3SSs (Egan et al., 2014) of which the prototypic systems are found in *Salmonella* Spp. and *Shigella flexneri*. The *Salmonella* and *Shigella* prototypic systems are required in these bacteria for host cell invasion and escape from the endocytic vacuole into the cytosol, respectively (reviewed in Galán et al., 2014). *B. pseudomallei* is a facultative intracellular pathogen capable of survival in both phagocytic and non-phagocytic cell lines (Jones et al., 1996). T3SS-3 is required for *B. pseudomallei* to efficiently escape the endocytic vesicle (Stevens et al., 2002). The T3SS-3 is also required for full virulence in both murine and Syrian hamster models of infection (Stevens et al., 2004; Warawa and Woods, 2005; Gutierrez et al., 2015a). T3SS-3 deficient mutants are also impaired in their ability to disseminate from the lungs of mice infected intra-nasally (Gutierrez et al., 2015a). A recent study used Tn-seq to identify genes required for respiratory melioidosis in mice and identified the following T3SS-3 genes as being required: *bprA* (BPSS1530), *bipC* (BPSS1531), *bipB* (BPSS1532), *bicA* (BPSS1533), *bsaZ* (BPSS1534), *bsaW* (BPSS1537), *bsaV* (BPSS1538), *bsaO* (BPSS1545), *bsaM* (BPSS1547), *bsaL* (BPSS1548), *bsaK* (BPSS1549), and *bsaJ* (BPSS1550) (Gutierrez et al., 2015b). This highlights the importance of T3SS-3 in melioidosis.

## Transcriptional regulation of T3SS-3

The transcriptional regulation of T3SS-3 has been elucidated (Sun et al., 2010). At the top of the regulatory hierarchy is the gene *bspR* (BPSL1105), which, when disrupted led to a reduced level of expression of genes in the T3SS-3 locus as shown by microarray and real time PCR (Sun et al., 2010). *bspR* signals through the membrane bound regulator *bprP* (BPSS1553) which controls expression levels of both structural and secreted components of T3SS-3 (Sun et al., 2010). *bprP* further signals through *bsaN* (BPSS1546) and its co-activator, the chaperone *bicA* (BPSS1533), controlling transcription of the known effectors *bopC, bopE* and *bopA*, as well as the chaperone *bicP* (BPSS1523) and the regulators *bprB-D* (BPSS1520-22) (Sun et al., 2010; Chen et al., 2014). *bsaN* also relays the regulation signal to the virulence-associated Type 6 secretion system and virulence factors such as *bimA* and *virAG* (Sun et al., 2010; Chen et al., 2014).

The presence of genes allowing for arabinose assimilation was one of the first methods used to differentiate between virulent *B. pseudomallei* and avirulent *B. thailandensis* (Smith et al., 1997; Moore et al., 2004). Expressing the *B. thailandensis* arabinose assimilation operon in *B. pseudomallei* causes a down-regulation of expression of the T3SS-3 genes, notably the T3SS regulator *bsaN*, and also results in a reduction in virulence in a Syrian hamster model of infection (Moore et al., 2004). This suggests that loss of the arabinose assimilation operon may account for some of the differential virulence observed between these two species of *Burkholderia* (Moore et al., 2004).

## Structure of T3SS-3

**Figure 2:**
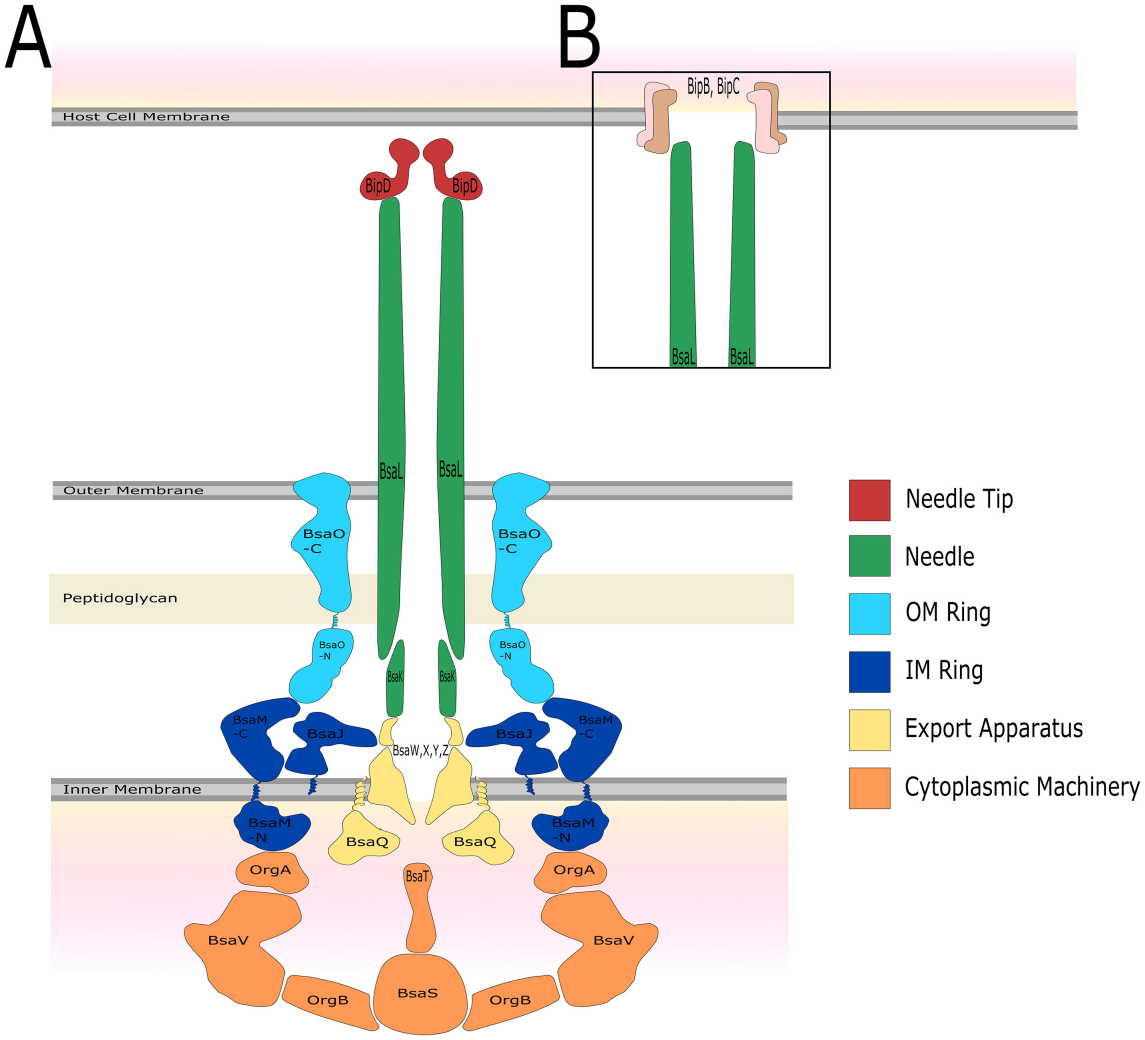
The predicted structure of the *B*. pseudo*mallei* K96243 T3SS-3 based on *Salmonella* SPI-1. The name of the protein which is predicted to constitute each structural component, as well as colors to represent different portions of the T3SS structure, are shown. **(A)** Before host cell contact, the T3SS is fully assembled spanning both the inner and outer bacterial membrane. The needle forms a channel extending out of the bacterium which is capped by the needle tip protein. **(B)** After contact with the host cell, a signal is relayed through the T3SS and translocator proteins form a pore in the host cell membrane allowing for injection of bacterial effector proteins.

The structure of the T3SS is well conserved and is similar to that of the bacterial flagella system (Kubori et al., 1998; Young et al., 1999; Gophna et al., 2003). It is thought that the flagella and the T3SS evolved from a similar ancestor, but the T3SS has been the product of a large amount of horizontal gene transfer (Gophna et al., 2003). T3SSs are separated into seven different families named after the archetype system in each family, each with slight differences in structure and host cell target, with multiple types of T3SS present in some bacterial species (e.g., plant or animal) (reviewed by Büttner, 2012). In recent years the structure of the *Salmonella* T3SS has been solved using cryo-EM (Schraidt and Marlovits, 2011) and cryo-ET (Hu et al., 2017). The structure of the *Salmonella* T3SS (reviewed by Galán et al., 2014) consists of inner and outer membrane rings connected by a rod, the extracellular needle and a secreted translocation pore which spans the target cell membrane. Assembly of the T3SS appears to be hierarchical and is the subject of multiple recent reviews (Büttner, 2012; Diepold and Wagner, 2014). Based on evidence from *Yersinia* and *Salmonella*, it appears construction of the T3SS begins with the formation of the outer-membrane/structural ring (Secretin) and independently the inner membrane export machinery, which are then linked by YscJ (*Yersinia*) or PrgK (*Salmonella*), followed by assembly of the ATPase/C-ring and the formation of the mature needle complex (Diepold et al., 2010, 2011; Wagner et al., 2010). The structural proteins in the *B. pseudomallei* T3SS-3 (Figure 2) that have been studied specifically are described below.

### Components of the export apparatus

#### BsaZ (BPSS1534)

BsaZ (BPSS1534) is a structural component with homology to SpaS of *Salmonella* (Stevens et al., 2002). SpaS is a component of the export apparatus and has been shown to undergo auto-cleavage causing a switch between secretion of structural components (early substrates) to secretion of the needle complex and translocator proteins (intermediate substrates) (Zarivach et al., 2008). *B. pseudomallei bsaZ* mutants are unable to secrete effector and translocator proteins (Stevens et al., 2003; Muangman et al., 2011; Vander Broek et al., 2015). Mutations in *bsaZ* result in a significant delay in escape from the phagosome and reduced intracellular survival in J774.2 cells (Stevens et al., 2002; Burtnick et al., 2008). *bsaZ* mutants are also attenuated in murine (Stevens et al., 2004; Burtnick et al., 2008) and Syrian hamster (Warawa and Woods, 2005) models of melioidosis. *bsaZ* mutants have proven to be a useful tool in understanding the role of T3SS-3 in *B. pseudomallei* pathogenesis, however, the effect of these mutations other than an inability to secrete effector proteins is not well understood. For example, it is unclear if BsaZ undergoes auto-cleavage and is involved in secretion hierarchy in a similar manner to SpaS. It has also not been determined if *bsaZ* mutants still assemble the external needle appendage, or at what stage in formation of the mature T3SS complex they are impaired.

#### BsaQ (BPSS1543)

Another component of the export apparatus of the *B. pseudomallei* T3SS-3 that has been described is BsaQ (BPSS1543), which is homologous to InvA from *Salmonella* (Sun et al., 2005). InvA is required for the formation of the mature T3SS in *Salmonella* as well as secretion of effector proteins (Kubori et al., 2000). *bsaQ* mutants display a similar phenotype to other T3SS-3 structural mutants in that they are delayed in phagosome escape, resulting in reduced intracellular survival (Muangsombut et al., 2008). The *bsaQ* mutants are unable to secrete the effector protein BopE or translocator tip protein BipD, and show significant defects in cell invasion (Muangsombut et al., 2008). Similarly to BsaZ, there is a gap in our knowledge of whether BsaQ truly functions in a manner similar to InvA.

### Inner membrane ring

#### BsaM (BPSS1547)

The inner membrane component BsaM (BPSS1547) is homologous to *Salmonella* PrgH, which forms the inner membrane ring of the *Salmonella* T3SS and is required for secretion of effector proteins (Kubori et al., 2000; Marlovits et al., 2004). A *B. pseudomallei bsaM* mutant induced lower levels of NF-κB signaling in HEK 293T cells when compared to the isogenic parent strain (Teh et al., 2014). The *bsaM* mutant activated NF-κB at time points corresponding to a delayed escape from the phagosome, suggesting it has similar effects to the other T3SS-3 structural knockouts (Teh et al., 2014).

### Needle components

#### BsaL (BPSS1548)

A crystal structure of the protein BsaL (BPSS1548) demonstrated significant structural similarity with MxiH (*Shigella*) and PrgI (*Salmonella*), which form the external needle structure (major needle subunit) of the T3SS (Zhang et al., 2006; Wang et al., 2007; Barrett et al., 2008). BsaL is recognized by the host cell neuronal inhibitory protein (NAIP) leading to activation of the NLRC4 inflammasome (Yang et al., 2013). NLRC4 has been shown to be important in a murine model of respiratory melioidosis and a human *NLRC4* polymorphism is associated with survival in melioidosis patients (West et al., 2014).

#### BsaK (BPSS1549)

The cellular protein Nod-like receptor NLRC4 recognizes T3SS minor needle component proteins from a range of Gram-negative bacteria, including BsaK (BPSS1549) from *B. pseudomallei*, activating an innate immune response through casapase-1 (Miao et al., 2010; Zhao et al., 2011; Bast et al., 2014). A *B. pseudomallei bsaK* mutant was highly attenuated in an intranasal murine model of melioidosis (Bast et al., 2014).

### Non-structural proteins

#### BsaU (BPSS1539)

BsaU (BPSS1539) is homologous to InvJ from *Salmonella* which is the “molecular ruler” which determines the length of the T3SS needle (Kubori et al., 2000). Mutation of *bsaU* in *B. pseudomallei* resulted in delay in phagosome escape and reduced virulence in a BALB/c intranasal mouse infection model (Pilatz et al., 2006). The mutant was also deficient in its ability to secrete the effector protein BopE and the translocator tip protein BipD (Pilatz et al., 2006). It can be hypothesized that without BsaU, the needle complex of T3SS-3 forms incorrectly, accounting for the lack of effector and translocator secretion.

#### BsaP (BPSS1544)

A family of T3SS proteins called the “gatekeeper” proteins (InvE/MxiC/SepL/YopN-TyeA) are involved in the control of effector and translocator protein secretion and the switch from intermediate to late substrates for secretion in *Salmonella*/*Shigella*/*E. coli*/*Yersinia*, respectively (reviewed in Büttner, 2012). These proteins are involved in the temporal regulation of their respective T3SSs and deletion of all of these proteins causes an increase in levels of secreted effectors, but has differing effects on the secretion of translocators. For example deletion of *invE* (Kubori and Galán, 2002) and *sepL* (Kresse et al., 2000; Deng et al., 2004, 2005) causes a reduction in translocator secretion, deletion of *mxiC* (Botteaux et al., 2009) has no effect on translocator secretion, and deletion of *yopN* (Forsberg et al., 1991; Iriarte et al., 1998) increases levels of secreted translocators. The closest homolog to this family of proteins in *B. pseudomallei* is BsaP (BPSS1544), which we have demonstrated functions as a gatekeeper protein for effectors in a manner most similar to *Salmonella invE* (Vander Broek et al., 2015). Deletion of *bsaP* creates a phenotype in which effector proteins are hyper-secreted with a concomitant decrease in translocator secretion (Vander Broek et al., 2015). Further studies are warranted to determine the molecular interactions of BsaP with other components of the T3SS-3, in order to fully understand BsaP in the context of the other members of the gatekeeper family of proteins.

### Structural components for which no data is available

Proteins for which no published work is available are OrgA (BPSS1551), OrgB (BPSS1552), BsaJ (BPSS1550), BsaO (BPSS1545), BsaT (BPSS1540), BsaV (BPSS1538), BsaW (BPSS1537), BsaX (BPSS1536), and BsaY (BPSS1535). The putative function of these proteins can be found in Table 2 and their predicted location in the structure of T3SS-3 is shown in Figure 2.

**Table 2:** *B. pseudomallei* T3SS-3 genes, corresponding proteins and predicted functions.

***B. pseudomallei*** **K96243**		**Homologs**		**Universal nomenclature**	**Function**
**T3SS-3**		***Salmonella* SPI-1**	***Shigella***	**Protein name**	
**Locus tag**	**Protein name**	**Protein name**	**Protein name**		
BPSS1516	BopC				Effector
BPSS1517	—				Chaperone of BopC
BPSS1518	—				
BPSS1519	—				
BPSS1520	BprC				Regulator of T6SS-1
BPSS1521	BprD				Effector/Regulator
BPSS1522	BprB				Putative regulator
BPSS1523	BicP	SicP			Chaperone, shown to bind to BapA
BPSS1524	BopA	SptP	IcsB		Effector protein
BPSS1525	BopE	SopE			Effector protein
BPSS1526	BapC	IagB	IpgF		Effector protein
BPSS1527	BapB	IacP			Negative regulator, predicted chaperone
BPSS1528	BapA				Effector protein
BPSS1529	BipD	SipD	IpaD	SctA	Needle tip
BPSS1530	BprA				Putative regulator
BPSS1531	BipC	SipC	IpaC	SctB	Minor translocator
BPSS1532	BipB	SipB	IpaB	SctE	Major translocator
BPSS1533	BicA	SicA	IpgC		Chaperone/Co-activator of BsaN
BPSS1534	BsaZ	SpaS	Spa40	SctU	Autoprotease, early/middle substrate switch
BPSS1535	BsaY	SpaR	Spa29	SctT	Inner membrane export apparatus
BPSS1536	BsaX	SpaQ	Spa9	SctS	Inner membrane export apparatus
BPSS1537	BsaW	SpaP	Spa24	SctR	Inner membrane export apparatus
BPSS1538	BsaV	SpaO	Spa33	SctQ	Cytoplasmic ring
BPSS1539	BsaU	InvJ	Spa32	SctP	Needle length control protein
BPSS1540	BsaT	InvI	Spa13	SctO	Stalk protein
BPSS1541	BsaS	InvC	Spa47	SctN	ATPase
BPSS1542	BsaR	InvB	Spa15		Chaperone, predicted to bind BopE
BPSS1543	BsaQ	InvA	MxiA	SctV	Export gate
BPSS1544	BsaP	InvE	MxiC	SctW	Middle/late substrate switch
BPSS1545	BsaO	InvG	MxiD	SctC	Outer membrane ring / secretin
BPSS1546	BsaN	InvF	MxiE		Regulator of T3SS-3 effector proteins
BPSS1547	BsaM	PrgH	MxiG	SctD	Inner membrane ring component
BPSS1548	BsaL	PrgI	MxiH	SctF	Outer rod/ major needle component
BPSS1549	BsaK	PrgJ	MxiI	SctI	Inner rod/minor needle component
BPSS1550	BsaJ	PrgK	MxiJ	SctJ	Inner membrane ring component
BPSS1551	OrgA	OrgA	MxiK	SctK	ATPase cofactor
BPSS1552	OrgB	OrgB	MxiN	SctL	Stator protein
BPSS1553	BprP				Regulator of T3SS-3
BPSS1554	BprQ				Regulator of T3SS-3

*Homologs from Salmonella SPI-1 and Shigella are given for reference. Where applicable, the universal nomenclature for T3SS structural proteins is also listed*.

T3SS-3 has been well studied in terms of its role in virulence however very little has been reported on the structure and the molecular interactions between individual components of the T3SS itself. Determining the finer points of structure and hierarchy of assembly represent a major unanswered research question in terms of understanding T3SS-3 in the context of other well studied T3SSs.

## Energizing secretion and unfolding of substrates

Proteins are secreted in an unfolded state due to the narrow width of the needle channel which is 2–3 nm in S. *flexneri* (Blocker et al., 2001; Radics et al., 2014). This was proven by cryo-EM imaging of a substrate which was engineered to become trapped in the secretion channel (Radics et al., 2014). The ATPase at the base of the secretion system has been shown to dissociate proteins from their chaperones and is also thought to be involved in unfolding of the protein (Akeda and Galán, 2005). The source of energy for exporting proteins is the subject of some debate. In the flagellar T3SS of *Yersinia* and *Salmonella*, secretion can take place in the absence of the ATPase (Wilharm et al., 2004; Erhardt et al., 2014). This evidence, combined with a study which showed flagellar T3S was halted in the absence of a proton gradient, has led to the hypothesis that the ATPase is primarily required for protein unfolding and that proton motive force energizes secretion of the unfolded substrate (Paul et al., 2008).

The *B. pseudomallei* T3SS-3 ATPase is BsaS (BPSS1541). A *B. pseudomallei bsaS* mutant was unable to secrete the known effector protein BopE, demonstrated a defect in intracellular survival in RAW264.7 cells and was attenuated in BALB/c mice infected intra-nasally, demonstrating that the ATPase is required for T3S of effector proteins and has a similar phenotype to other T3SS-3 structural mutants (Gong et al., 2015). This agrees with a previous study in which *B. pseudomallei* and *B. thailandensis* containing in-frame deletions in *bsaS*, were impaired in their ability to escape the endosomal compartment and form plaques in host cell monolayers as a result of cell-to-cell spread (French et al., 2011). *Salmonella* lacking a functional ATPase will still assemble a mature T3SS, and will still secrete effector proteins, though at a reduced level (Erhardt et al., 2014). The effect that a *bsaS* mutation in *B. pseudomallei* T3SS-3 may have on either assembly of the T3SS apparatus or the secretion of early/middle substrates, such as the major needle component (BsaL) and the needle tip protein (BipD) remains an interesting open question.

## Needle tip and translocator proteins-sensing host contact

Upon host cell contact, the translocators of the T3SS are inserted into the host cell membrane forming a pore through which effector proteins may pass. *Shigella* is able to lyse red blood cells by inserting the translocators IpaB and IpaC into the RBC membrane forming a 25 angstrom pore (Blocker et al., 1999). Secretion of the translocator proteins is controlled by the needle tip protein. A *Shigella* needle tip protein mutant (*ipaD*), constitutively secreted the translocators IpaB and IpaC (Picking et al., 2005). Immunofluorescence microscopy demonstrated the presence of the *Shigella* IpaD protein on the surface of the bacteria in the absence of a host cell membrane, a finding that was further confirmed by electron microscopy (Espina et al., 2006). The needle tip protein probably acts to sense host cell contact, inhibiting premature secretion of translocators (Espina et al., 2006). In the same study it was shown that antibodies to the tip protein disrupted the haemolysis of sheep erythrocytes indicating the functional importance of IpaD in the insertion of the translocator complex into eukaryotic cell membranes, as well as regulating effector protein secretion (Espina et al., 2006). Next, the T3SS senses host cell contact when IpaD binds to bile salts causing IpaB to be exposed at the needle tip (Olive et al., 2007; Stensrud et al., 2008).

The final step in secretion of the translocation pore is dependent on *Shigella* interacting with host cell lipids to induce IpaC secretion (Epler et al., 2009). When cultured in the presence of liposomes, IpaC localizes to the bacterial surface and is secreted (Epler et al., 2009). This agrees with earlier evidence that cholesterol is bound by translocation components (*Salmonella* SipB and *Shigella* IpaB), is important for entry into host cells and is required for efficient translocation of effector proteins into host cells (Lafont et al., 2002; Hayward et al., 2005). There is some evidence to suggest that the translocator proteins, along with the needle tip and a functional T3SS, may be sufficient to determine the intracellular niche of *Salmonella* and *Shigella* in the absence of any of the effector proteins (Du et al., 2016).

### BipD (BPSS1529)

BipD (BPSS1529) is the needle tip protein of the *B. pseudomallei* T3SS-3. It is homologous to SipD (*Salmonella*), IpaD (*Shigella*), and LcrV (*Yersinia*). BipD has been confirmed to be secreted by the *B. pseudomallei* T3SS-3 (Stevens et al., 2003; Vander Broek et al., 2015). The crystal structure of BipD was solved (Erskine et al., 2006; Knight et al., 2006; Roversi et al., 2006; Johnson et al., 2007; Pal et al., 2010) and the 3-dimensional structure is highly similar to IpaD of *Shigella* (Johnson et al., 2007) and *Salmonella* SipD (Espina et al., 2007), but *bipD* cannot functionally complement *sipD* in *Salmonella* (Klein et al., 2017). It was demonstrated that the structure of BipD, as well as IpaD and SipD, is dependent on pH changes (Markham et al., 2008). Interestingly when cultured in a more acidic pH of 4.5, *B. thailandensis* secretes increased amounts of BipD as well as BopE (Jitprasutwit et al., 2010), though the study also described amino acid differences between BipD of *B. pseudomallei* and *B. thailandensis* (Jitprasutwit et al., 2010), raising the question as to what effect pH may have on T3SS-3 in *B. pseudomallei*.

In a *B. pseudomallei bipD* mutant, the levels of both translocators and effectors secreted into the culture supernatant are increased (Stevens et al., 2003; Vander Broek et al., 2015), in agreement with data published for the homologous *Shigella* protein IpaD (Parsot et al., 1995; Picking et al., 2005). IpaD blocks secretion of effector proteins until host cell contact has taken place (Roehrich et al., 2013), and because of its similarity in both sequence and structure (Erskine et al., 2006) it is perhaps unsurprising that BipD would function in a similar manner. More recent evidence suggests that *Shigella* IpaD may be involved in controlling the secretion of translocator and effector proteins through an interaction with the gatekeeper protein MxiC (Roehrich et al., 2016). This would suggest an interaction between BipD and BsaP in *B. pseudomallei* which may have a similar activity.

Needle tip proteins are of particular interest because of their potential use as subunit vaccines. Most notably the *Yersinia pestis* needle tip protein LcrV (V antigen), especially when combined with the Fraction 1 (F1) protein, is an effective vaccine that has been tested in human clinical trials (reviewed by Williamson, 2009). Vaccination of mice with the *Shigella* needle tip protein IpaD, along with the translocator IpaB, induced high levels of protection upon subsequent challenge (Martinez-Becerra et al., 2012, 2013; Heine et al., 2013). Some 15 years ago, it was described that convalescent serum from a melioidosis patient reacted specifically with a recombinant GST-tagged BipD protein (Stevens et al., 2002). CD4+ T cells taken from mice infected with an attenuated strain of *B. pseudomallei* showed specificity for BipD (Haque et al., 2006). Similarly, human monocyte-derived dendritic cells from healthy *B. pseudomallei* seropositive donors were pulsed with purified BipD after which CD4+ T cells were able to recognize the recombinant protein (Tippayawat et al., 2011). A *B. pseudomallei bipD* mutant was significantly attenuated in a BALB/c intranasal murine infection model, demonstrating the importance of this protein *in vivo* (Stevens et al., 2004). Attempts have been made to use BipD as a recombinant subunit vaccine in a BALB/c murine intraperitoneal model of infection, but in both studies the vaccine showed no protection upon subsequent challenge (Stevens et al., 2004; Druar et al., 2008).

### BipB (BPSS1532)

BipB (BPSS1532) shares 46% amino acid identity with the *Salmonella* translocator protein SipB, and is secreted by T3SS-3 in a *bsaZ* dependant manner (Vander Broek et al., 2015). In a *Salmonella sipB* mutant, *bipB* is unable to complement *sipB* demonstrating evolutionary separation of these proteins (Klein et al., 2017). This is an important reminder that although T3SSs are similar in structure, T3SS proteins do not always function identically.

A *B. pseudomallei* K96243 *bipB* insertion mutant showed reduced invasion and cell-to-cell spread in HeLa cells, and a reduced ability to form multi-nucleated giant cells in J774 cells (Suparak et al., 2005). *In vivo*, in BALB/c mice infected intra-nasally, the *bipB* mutant was greatly attenuated and showed a phenotype similar to that of the *bipD* translocator mutant (Stevens et al., 2004; Suparak et al., 2005). This is likely due to the inability of the T3SS to function correctly without the formation of the translocation pore, and any proposed secondary function would require further investigation. The N-terminal region of BipB has been tested as a protective antigen in a murine model of melioidosis, but as with BipD, showed no protection upon subsequent challenge (Druar et al., 2008).

### BipC (BPSS1521)

BipC (BPSS1531) is a homolog of the *Salmonella* SipC and *Shigella* IpaC translocator proteins. The *Salmonella* SipC protein has been shown to interact with SipB to form the translocon pore (Myeni et al., 2013). Beyond their role as a translocator protein, SipC and IpaC also function as effector proteins within the eukaryotic cell. SipC has actin nucleation activity and bundles F-actin (Hayward and Koronakis, 1999). The ability of SipC to nucleate and bundle F-actin as well as form the translocation pore, are all dependant on the C-terminal 209 amino acids of the 409 amino acid protein (Hayward and Koronakis, 1999; Chang et al., 2005; Myeni and Zhou, 2010). Actin bundling appears to be important for cell invasion, as *Salmonella* containing a *sipC* mutation abolishing its actin bundling activity, but not translocator function, was less invasive than the parental strain (Myeni and Zhou, 2010). Internalization of *B. pseudomallei* can be blocked by the actin polymerization inhibitor cytochalasin D (Jones et al., 1996), indicating the importance of actin cytoskeletal rearrangements in the uptake of *B. pseudomallei*. Similarly to SipC, BipC is also able to polymerise actin *in vitro* and stabilizes F-actin indicating possible actin bundling activity (Kang et al., 2016a and our own unpublished observations). Interestingly, *Salmonella* SipA protein enhances the ability of SipC to nucleate and bundle F-actin (McGhie et al., 2001), but a homolog of SipA is not encoded by the genome of *B. pseudomallei*. SipC has also been shown to bind to host Syntaxin 6 and thereby recruit LAMP1 to the *Salmonella* containing vacuole (SCV), helping to stabilize its membrane (Madan et al., 2012). The functional relevance of the actin-binding activity of BipC and whether it binds any other host cell proteins requires further study.

In *Salmonella* and *Shigella*, the SipC/IpaC family of translocator/effector proteins may play a crucial role in determining the intracellular niche of the bacteria. There is evidence to suggest that *sipC* and *ipaC* cannot fully complement each other (Osiecki et al., 2001; Klein et al., 2017). The authors suggest the proteins, while both translocators, may have some divergent functions which may parallel the different intracellular lifestyles of the pathogens, with *Salmonella* residing within the vacuole and *Shigella* rapidly escaping into the cytosol (Osiecki et al., 2001). This is supported by a recent study in which *Salmonella* expressing *ipaC*, was shown to be capable of vacuole escape (Du et al., 2016). Because of closer parallels between the intracellular lifestyles of *B. pseudomallei* and *Shigella*, we would predict that BipC would function in a manner more similar to IpaC than SipC, mediating the exit of the bacterium from the endocytic compartment into the host cell cytosol. In a *Salmonella sipC* mutant, *bipC* is unable to complement *sipC* (Klein et al., 2017), further supporting a possible difference in accessory function of these proteins.

The C-terminal and N-terminal regions of BipC have previously been separately tested as a protective antigen in mice, but neither antigen showed any protection upon challenge with *B. pseudomallei* (Druar et al., 2008). In another study, a *B. pseudomallei bipC* mutant showed reduced cell adhesion and invasion of A549 cells (Kang et al., 2015). This *bipC* mutant also showed a delay in escape from the phagosome, leading to a delay in formation of actin tails in the host cell cytoplasm and intracellular replication (Kang et al., 2015). The mutant was also attenuated in BALB/c mice infected intraperitoneally (Kang et al., 2015). The transcriptome of livers of mice infected with the *bipC* mutant showed a lower expression of genes involved in actin cytoskeleton signaling, MAPK signaling, integrin signaling and TNF when compared to mice infected with the parental *B. pseudomallei* strain (Kang et al., 2016b). While this shows the importance of BipC in virulence, both *in vivo* and *in vitro*, it does not separate the role of BipC as a translocator necessary for a functional T3SS and rapid escape from the endosome, from its role as an effector protein.

## Secretion signals and chaperones

Type III secretion signals are located at the N-terminus of a protein, are not cleaved and do not share primary sequence identity with each other (Michiels and Cornelis, 1991; Schesser et al., 1996). In *Yersinia*, it was demonstrated that as little as 15 amino acids of the N-terminus of YopE were required for secretion (Sory et al., 1995). Interestingly, substituting the alanines at position 2 and 15 in the YopE secretion signal with glutamic acid, did not affect secretion (Anderson and Schneewind, 1997). Even shifting the reading frame did not prevent the secretion of YopE, which the authors suggested may indicate a secretion signal located in the mRNA encoding the effector protein (Anderson and Schneewind, 1997).

A second important signal in the N-terminal region of T3SS effector proteins is the chaperone binding domain (CBD) which is required for the specific secretion of effector proteins (Abe et al., 1999; Ehrbar et al., 2003, 2006; Lee and Galán, 2003, 2004) as well as for their stability in the bacterial cytoplasm (Frithz-Lindsten et al., 1995; Abe et al., 1999). By determining the crystal structure of an effector protein bound to its chaperone, it is known that T3SS chaperones in *Yersinia* and *Salmonella* bind the N-terminal amino acids of their effector protein, just after the T3S signal, but before any functional domains, and maintain the bound effector protein in a partially unfolded state that may be more competent for secretion (Birtalan and Ghosh, 2001; Stebbins and Galán, 2001).

The T3S signal also appears to be promiscuous between systems, allowing secretion of effector proteins from bacteria in an unrelated host bacterium containing a T3SS (Rossier et al., 1999; Hovis et al., 2013). This is even true of less similar T3SSs, as demonstrated by the ability of the Hrp Plant T3SS of *Xanthomonas* to secrete the mammalian effector protein *Yersinia* YopE (Rossier et al., 1999). Perhaps because of the similarity between the two, virulence-associated T3SS effectors can be secreted by the bacterial flagellar T3SS system in *Yersinia* and *Salmonella* (Young and Young, 2002; Lee and Galán, 2004; Warren and Young, 2005; Ehrbar et al., 2006). It is only through interaction with the appropriate chaperone that specific secretion through a single system is achieved. Inhibition of the ability of *Salmonella* SopE to bind its chaperone InvB, caused secretion through both the flagellar and SPI-1 virulence associated T3SS (Lee and Galán, 2004; Ehrbar et al., 2006). The genome of *B. pseudomallei* encodes five putative T3SS-3 chaperones, though little work concerning their function has yet been described.

### BPSS1517

The protein encoded by the gene BPSS1517 is predicted to be a putative chaperone (Panina et al., 2005) and has been shown to interact with the downstream effector protein BopC (BPSS1516) (Muangman et al., 2011).

### BicP (BPSS1523)

BicP (BPSS1523) shares homology with *Salmonella* SicP, a specific chaperone for the effector protein SptP (Fu and Galán, 1998). The closest homolog to SptP in *B. pseudomallei* is BopA (BPSS1524) which was predicted to be the binding partner for BicP (Panina et al., 2005), but the interaction between the two proteins has not formally been demonstrated.

### BapB (BPSS1526)

BapB (BPSS1526) is homologous to *Salmonella* IacP (Stevens et al., 2002). IacP is important for *Salmonella* invasion into host cells by playing a role in regulating SopA, SopB and SopD secretion (Kim et al., 2011). Formerly considered a possible candidate effector protein, BapB was not found to be secreted by T3SS-3 (Treerat et al., 2015; Vander Broek et al., 2015). Deletion of *bapB* caused an increase in the transcription and secretion of BopE, indicating it may be a negative regulator of effector transcription (Treerat et al., 2015). In the same study the authors performed a phylogenetic analysis which suggested that BapB may be closely related to the *Salmonella* FliT chaperone protein (Treerat et al., 2015).

### BicA (BPSS1533)

BicA (BPSS1533) is homologous to the *Salmonella* chaperone SicA. SicA binds to and prevents the association and resulting degradation of SipC and SipB in the bacterial cytoplasm (Tucker and Galán, 2000). As SipC is secreted by a mature needle complex, SicA is freed and interacts with the transcriptional regulator InvF to increase the expression of effector proteins (Tucker and Galán, 2000; Darwin and Miller, 2001). *B. pseudomallei* BicA is required for the secretion of the known effector proteins BopE and BopA (Sun et al., 2010), and BicA along with the regulator BsaN activate transcription of T3SS-3 effector proteins, translocators and chaperones (Chen et al., 2014). The *bicA* gene is also able to partially complement a *Salmonella sicA* mutant (Klein et al., 2017), further supporting a homologous function. BicA is required for respiratory melioidosis in mice (Gutierrez et al., 2015b).

### BsaR (BPSS1542)

BsaR (BPSS1542) is homologous to the *Salmonella* chaperone InvB which has been shown to be required for secretion of the effector proteins SopE and SopA (Ehrbar et al., 2003, 2004; Lee and Galán, 2003). BsaR is predicted to be the chaperone for BopE using a computational screen for chaperones in *B. pseudomallei* K96243 (Panina et al., 2005), although this has not yet been experimentally validated.

## T3SS-3 effector proteins

The role of the T3SS is to deliver an array of effector proteins into the target cell to subvert host cell functions. The function of different effector proteins is extremely varied, ranging from blocking apoptosis (*E. coli* NleH) (Hemrajani et al., 2010), prevention of phagocytosis (*Yersinia* YopH) (Persson et al., 1997), cytotoxic activity (*Pseudomonas aeruginosa* ExoU) (Sato et al., 2003) and disruption of the actin cytoskeleton (*Salmonella* SopE) (Hardt et al., 1998). *B. pseudomallei* encodes seven effector proteins known to be secreted by T3SS-3 (CHBP, BopC, BopA, BapA, BprD, BapC, and BopE), as well as one hypothetical T3SS effector BopB (Pumirat et al., 2014; Vander Broek et al., 2015).

### CHBP/Cif (BPSS1385)

CHBP (BPSS1385) is a homolog of *E. coli* cell cycle inhibiting factor (Cif) and is the only identified T3SS-3 effector protein that is encoded outside of the T3SS-3 locus. Cif and CHBP are able to deamidate cellular NEDD8 causing cell cycle arrest (Nougayrède et al., 2001; Cui et al., 2010) and CHBP causes cell cycle arrest when expressed in *B. thailandensis* (Cui et al., 2010). CHBP has also been shown to activate the cellular kinase ERK independent of its ability to deamidate NEDD8 (Ng et al., 2017).

The gene encoding CHBP is present in ~76% of the available *B. pseudomallei* genomes and a Western blot assay used to probe for the presence of the CHBP protein in *B. pseudomallei* clinical isolates from the endemic region detected CHBP in 47% of isolates tested (Pumirat et al., 2014). Interestingly, CHBP is not secreted under standard growth conditions, but *B. pseudomallei* secretes CHBP in U937 cells in a *bsaQ*–dependent manner (Pumirat et al., 2014). A *B. pseudomallei* K96243 *chbP* insertion mutant was impaired in its ability to form plaques in HeLa cells at 24 h and demonstrated lower cytotoxicity at 6 h as assessed by LDH release assays (Pumirat et al., 2014). Both phenotypes could be complemented by expression of *chbP in trans* indicating that these phenotypes were due to disruption of the *chbP* gene and not due to unexpected polar effects of the insertion mutation (Pumirat et al., 2014).

### BopB/FolE (BPSS1514)

BopB (BPSS1514) or FolE, is annotated to be a GTP cyclohydrolase I and was thought to be a candidate effector protein (Stevens et al., 2004). Yet a *bopB* mutant did not display a significantly reduced time to death in a BALB/c intraperitoneal infection model (Stevens et al., 2004). Similarly, mutation of *bopB* did not affect invasion and intracellular replication of host cells (Chen et al., 2014). Expression of *bopB* is co-regulated with the other T3SS-3 effectors by BsaN, but the role BopB plays in infection is still unknown (Chen et al., 2014).

### BopC (BPSS1516)

BopC (BPSS1516) is a 509 amino acid protein with no significant sequence homology to proteins from other species besides *B. mallei*. It is encoded just before the T3SS-3 locus along with BPSS1517, its chaperone (Muangman et al., 2011). BopC was detected in the culture supernatants of WT *B. pseudomallei* 10276, but not in supernatants of a *bsaZ* insertion mutant, indicating that BopC is secreted by T3SS-3 (Muangman et al., 2011; Vander Broek et al., 2015). The first 20 amino acids of BopC fused to the β-lactamase gene TEM1, was sufficient for translocation into HeLa cells in a T3SS-dependant manner (Muangman et al., 2011). A *B. pseudomallei* K96243 *bopC* mutant was hindered in its ability to invade A549 cells (Muangman et al., 2011) and displayed reduced levels of intracellular survival (Srinon et al., 2013). The *bopC* mutant also demonstrated delayed phagosome escape in J774A.1 cells as shown by staining for co-localisation of the bacteria with the cellular lysosomal marker protein LAMP–1, which likely explains the defect in intracellular survival (Srinon et al., 2013).

### BprD (BPSS1521)

BprD (BPSS1521) has no known homology to proteins outside of *B. pseudomallei* and the closely related *Burkholderia* species. It is labeled as a putative regulator of T3SS-3, though a knockout of the *bpr* operon (*bprB-D*) showed no effect on the expression of T3SS-3 genes (Sun et al., 2010). Its expression is regulated by BsaN along with the known effector proteins BopA, BopC and BopE (Sun et al., 2010; Chen et al., 2014) and *bipD* gene expression is significantly up-regulated in tissues of infected mice (Chirakul et al., 2014). The same study demonstrated attenuation of the *bprD* mutant in BALB/c mice infected intraperitoneally, which the authors speculate may be due to the up-regulation of the T6SS-1 through effects on *bprC* (Chirakul et al., 2014).

Our own work has demonstrated that BprD is secreted into the supernatant in a T3SS-3 *bsaZ*-dependant manner (Vander Broek et al., 2015). While this may seem surprising, it is not without precedent that a regulator of the T3SS is also a substrate for secretion, for example, *Yersinia* LcrQ (Cambronne et al., 2000). It is thought that LcrQ acts as a feedback inhibitor of the expression of *Yersinia* effectors (Cambronne et al., 2000). When LcrQ is secreted into host cells and the levels of LcrQ in the bacterium are depleted, inhibition is relieved and T3S can progress (Cambronne et al., 2000). Whether BprD is secreted into host cells, whether it acts as a true effector protein and the mechanisms by which it regulates virulence and T6S present interesting research questions for the field.

### BopA (BPSS1524)

BopA (BPSS1524) shares 23% amino acid identity with *Shigella* IcsB (Cullinane et al., 2008). It has been predicted to contain a Rho GTPase inactivation domain (RID) similar to that found in *Vibrio cholerae* VcRtxA and other MARTX toxins which indirectly inactivate Rho GTPases (Pei and Grishin, 2009). IcsB, along with its chaperone IpgA, are important for *Shigella*'s ability to escape LC3-positive autophagosomes once inside the host cell, and this activity is dependent on the IcsB cholesterol-binding domain (Kayath et al., 2010; Campbell-Valois et al., 2015). BopA also contains a functional cholesterol-binding domain (Kayath et al., 2010). The *B. pseudomallei* homolog of IpgA is BicP, which co-purifies with BopA and helps to prevent its degradation, indicating that it is the chaperone for BopA (Kayath et al., 2010). BopA is secreted by T3SS-3 in a *bsaZ*-dependant manner (Vander Broek et al., 2015) and the first 58 amino acids of *B. mallei* BopA fused to the *Yersinia enterolitica* phospholipase YplA, has been shown to be secreted in a surrogate enteropathogenic *E. coli* host (Whitlock et al., 2008).

BopA is important for the intracellular survival of *B. pseudomallei* in phagocytic cells (Cullinane et al., 2008). A *B. pseudomallei* K96243 *bopA* mutant displayed reduced intracellular survival and an increased localisation with GFP-LC3, an indicator of autophagy stimulation, in RAW 264.7 cells (Cullinane et al., 2008). This reduction in intracellular survival was overcome when cells were treated with the autophagy inhibitor wortmannin (Cullinane et al., 2008). Another study demonstrated that BopA is important for escape of the bacterium from the phagosome (Gong et al., 2011). A *B. mallei* ATCC 23344 *bopA* mutant demonstrated reduced intracellular survival in J774A.1 cells (Whitlock et al., 2008). Interestingly, in the murine alveolar macrophage cell line MH-S, the same *B. mallei bopA* mutant exhibited increased intracellular survival when compared to the isogenic parental strain, indicating that different cell types may rely on different mechanisms to control intracellular *B. mallei* (Whitlock et al., 2009). BALB/c mice infected intraperitoneally with a *B. pseudomallei* 576 *bopA* insertion mutant were significantly delayed in time to death when compared to the parental strain (Stevens et al., 2004). Similarly, BALB/c mice infected intra-nasally with a *B. mallei* ATCC 23344 *bopA* insertion mutant also showed a delayed time to death. No bacteria were recovered from the lung tissue of animals infected with the *bopA* mutant while 10^8^ bacteria were recovered from the lungs of animals infected with *B. mallei* (Whitlock et al., 2009). In mice immunized with recombinant BopA and challenged intra-nasally with *B. mallei* ATCC23344 or *B. pseudomallei* 1026b, the BopA vaccine protected 100 and 60%, respectively of animals 21 days post infection (Whitlock et al., 2010).

### BopE (BPSS1525)

BopE (BPSS1525) is the best characterized of the *B. pseudomallei* effector proteins and is commonly used as a readout for the ability of the T3SS-3 to secrete effector proteins. BopE is 27% identical over a region of 168 amino acids to the *Salmonella* guanine nucleotide exchange factor (GEF) SopE (Stevens et al., 2003). BopE is secreted by *B. pseudomallei* in a manner dependent on the T3SS-3 (Stevens et al., 2003; Muangsombut et al., 2008; Vander Broek et al., 2015) and is required for efficient invasion of non-phagocytic cells (Stevens et al., 2003). Also, ectopic expression of BopE in HeLa cells causes significant actin cytoskeletal rearrangements with similarity to ectopic SipC expression (Stevens et al., 2003). Using fluorescence spectrometry, it was demonstrated that purified BopE, similar to SopE, is a functional GEF for both Rac1 and Cdc42, but with about 10 fold lower activity than SopE (Stevens et al., 2003). This lower activity may be explained by differences in the catalytic domains, as the SopE catalytic domain stays in an open conformation, while BopE adopts a closed conformation which requires interaction with Cdc42 to allow for GEF activity (Upadhyay et al., 2008). HEK 293T cells transfected with plasmids expressing BopE and caspase–1, resulted in increased activation of caspase–1 and –7 (Bast et al., 2014). When the active site of the transfected BopE was mutated, levels of activation of caspase–1 and –7 returned to basal levels indicating BopE's GEF activity is required for the activation of caspase–1 and –7 (Bast et al., 2014).

BopE is a potent T cell antigen in mice (Haque et al., 2006) and in sero-positive recovered melioidosis patients (Tippayawat et al., 2009). However, in an intraperitoneal BALB/c murine model of melioidosis, ablation of the *bopE* gene did not affect the median time to death of the animals when compared to the parental strain (Stevens et al., 2004).

### BapC (BPSS1527)

BapC (BPSS1526) is homologous to *Salmonella* IagB (Stevens et al., 2002). IagB is thought to be a lytic transglycosylase involved in the breakdown of the bacterial peptidoglycan layer, allowing connection of the inner and outer membrane components of the secretion system, though this function has not been formally demonstrated (Zahrl et al., 2005). BapC is secreted by T3SS-3 in a manner dependant on *bsaS* (Treerat et al., 2015). A *B. pseudomallei* K96243 *bapC* mutant showed a slight attenuation in a competitive growth assay in an acute BALB/c model of infection (Treerat et al., 2015), whereas a *B. pseudomallei* 1026b *bapC* mutant showed no significant attenuation in a Syrian hamster model of melioidosis (Warawa and Woods, 2005).

### BapA (BPSS1528)

BapA (BPSS1528) has no known homology to any other bacterial or host cell proteins except the BapA orthologues of *B. pseudomallei* and the closely related species *B. thailandensis* and *B. mallei*. BapA is secreted by T3SS-3 in both a BsaS and BsaZ dependant manner (Treerat et al., 2015; Vander Broek et al., 2015). A 1026b *B. pseudomallei bapA* mutant showed no attenuation in a Syrian hamster model of infection (Warawa and Woods, 2005), whereas a *B. pseudomallei* K96243 *bapA* mutant was attenuated in a competitive growth assay in an acute BALB/c model of infection (Treerat et al., 2015).

## Future perspectives

While there has been a wealth of new information concerning *B. pseudomallei* T3S, there are still many significant gaps in knowledge, particularly with regard to the importance of T3SS-1 and T3SS-2 in melioidosis. It is possible that the plant pathogen-like T3SS-1 and T3SS-2 add fitness to *B. pseudomallei* in the environment, allowing infection/colonization of adjacent plant life. However, it is also possible that these systems are relevant in mammalian hosts, given their conservation amongst the *B. pseudomallei* strains sequenced to date, and the finding that T3SS-1 is required for full virulence in a murine model of melioidosis (D'Cruze et al., 2011).

It is still unclear whether T3SS-3 plays a significant role in invasion of host cells, particularly non-phagocytic cells. There have been many reports of T3SS-3 or its effector proteins playing a role in invasion (Stevens et al., 2003; Suparak et al., 2005; Muangsombut et al., 2008; Muangman et al., 2011; Kang et al., 2015). Yet in another study, T3SS-3 ATPase (*bsaS*) mutants did not show a decrease in invasion efficiency in HEK293 or HeLa cells (French et al., 2011). This observation is important because it questions the involvement of T3SS-3 in invasion as well as highlighting a general lack of understanding of the pathways and mechanisms involved in *B. pseudomallei* entry into host cells.

To date CHBP is the only effector protein secreted by the *B. pseudomallei* T3SS-3 that is encoded outside of the T3SS-3 locus, and which is not present in the genome of all strains (Pumirat et al., 2014). This raises the question of whether other effector proteins secreted by T3SS-3, but encoded outside of the T3SS-3 locus, may be present in other strains. Full genome comparisons of almost 100 strains of *B. pseudomallei* identified 86% of genes as being conserved and present in all strains (Sim et al., 2008). The other 14% of genes, considered accessory genes, were disproportionally present in genomic islands and were associated with clinical isolates (Sim et al., 2008). The GIs in *B. pseudomallei* are highly variable between strains. A study of five clinical *B. pseudomallei* strains identified a total of 71 GIs distributed between the strains, with at least half being unique to the strain in which they were identified (Tuanyok et al., 2008). The variability in these regions is largely due to horizontal gene transfer and *B. pseudomallei* has a relatively high rate of lateral gene transfer compared to the mutation rates of other bacterial species (Pearson et al., 2009). As *B. pseudomallei* has a large amount of genomic diversity in its accessory genome and a high rate of lateral gene transfer, there is a strong possibility that other novel effector proteins are encoded in other strains of *B. pseudomallei* that have yet to be identified. Study of these effector proteins could provide important insights into strain differences as well as the potential for novel effector biology, but it is a difficult task as the secretome of *B. pseudomallei* is very complex (Vander Broek et al., 2015). Also, due to the temporal and hierarchical control of the T3SS, there is always the possibility that an effector protein will not be secreted under the conditions used in a given experiment (reviewed in Büttner, 2012). Previous methods of identifying T3SS-3 effector proteins have relied on initial bioinformatics prediction (Stevens et al., 2003; Muangman et al., 2011) or high throughput screens (Vander Broek et al., 2015), both of which may prove to be useful tools for further studies of *B. pseudomallei* T3S. Indeed it is timely to apply these approaches to the identification and characterization of effector proteins secreted by the lesser studied T3SS-1 and T3SS-2.

Another important outstanding area of research is characterizing the functions of those effector proteins that have already been identified. One common characteristic of many T3SS effector proteins that complicates this task is their functional redundancy (reviewed in Galán, 2009). Commonly, deletion of one effector protein yields little or no phenotype in infection models because other effector proteins target either the same host cell protein or pathway (reviewed in Galán, 2009). In a biological system this would increase the chance of effectors successfully carrying out their intended function and decrease the likelihood of host cell interference. This functional redundancy also highlights the evolutionary importance of dysregulating specific cellular pathways from the standpoint of the bacterium. Even where redundancy does not exist, the function of effector proteins are often subtle when compared to bacterial toxins and may not be easily measurable in *in vitro* or *in vivo* models of infection (reviewed in Dean, 2011). This lack of a phenotype to inform focused studies presents a challenge for investigators, creating the need for high throughput “fishing” assays, such as protein immunoprecipitation/pull-downs and yeast two-hybrid assays. These assays have been used successfully to identify host cell binding partners and the subsequent functions of effector proteins from other bacteria (Zhou et al., 2013; Pallett et al., 2014). Understanding the functions of T3SS-3 effector proteins may provide new insights into host-pathogen interactions in *B. pseudomallei* infection.

Finally, the T3SS-3 has been shown to be one of the most important virulence factors in *B. pseudomallei* models of infection, raising the question of whether T3SS-3 could be a useful target for protective vaccines or therapeutic intervention in melioidosis patients. Although several attempts have been made to use live-attenuated vaccines based on mutation of key T3SS-3 genes in murine models of melioidosis, none have proven to provide sterilizing immunity. Despite being a potent B- and T-cell antigen, attempts to utilize BipD as a subunit vaccine in murine models of melioidosis have shown little promise (Stevens et al., 2004; Druar et al., 2008). There have also been attempts to use the translocator proteins of T3SS-3 as subunit vaccines. The N-terminal region of BipB was tested as a protective antigen in mice, but showed no protection against subsequent challenge (Druar et al., 2008). The C-terminal and N-terminal regions of BipC have also been separately tested as a subunit vaccine in mice, but neither antigen showed any protection (Druar et al., 2008). Some studies have focused on the use of effector proteins as subunit vaccines. Out of these studies the BopA protein shows most promise, since mice immunized with recombinant BopA were protected against subsequent intranasal challenge with both *B. mallei* and *B. pseudomallei* (Whitlock et al., 2010). More recently interest in the use of small molecule inhibitors of the T3SS-3 has arisen (Gong et al., 2015). Treatment of *B. pseudomallei* infected RAW264.7 cells with a small molecule inhibitor targeting BsaS of the T3SS-3, resulted in a decrease in bacterial intracellular survival (Gong et al., 2015). However, the use of such inhibitors is still very much in its infancy, with important *in vivo* studies being required to determine whether such small molecules would be effective in murine models of melioidosis.

## Author contributions

CV and JS contributed equally to the writing of this review article.

### Conflict of interest statement

The authors declare that the research was conducted in the absence of any commercial or financial relationships that could be construed as a potential conflict of interest.
